# Opening of a New Hospital at Sevenoaks

**Published:** 1902-10-25

**Authors:** 


### OPENING OF A NEW HOSPITAL AT SEVENOAKS.

On Thursday, October 16th, the new Hospital for Children suffering from Hip Disease was opened at Sevenoaks by the Lady Norah Hodgson. Some three hundred persons were present, most of whom were residents in the neighbourhood, and not all could find room in the large ward set apart for the ceremony. This consisted of a short service of dedication conducted by the Rev. H. Percy Thompson, Hon. Chaplain, and other clergy, while the singing of the hymns "Thine arm, O Lord, in days of old" and "There's a Friend for little children" was led by a surpliced choir.

### The Day of Small Things.

The Rev. H. P. Thompson briefly recalled the history of the hospital, which began 30 years ago, when Miss Jackson, the lady superintendent, took a little child into her house. A few years later a generous donation was made towards a building fund, and now nearly £12,000 has been subscribed, represented by the building and the site. The speaker mentioned several names which will always be gratefully remembered by all who have to do with the hospital—Hugh and Elizabeth Jackson, the founders; Henry Rose, who gave "not only his money but his daughter"; Mary Bevington, and Ethel Maud Sparrow, in whose memory a cot has been endowed—and concluded by reading a paragraph from a letter written by the mother of a former patient now serving in the 1st Duke of Cornwall's Light Infantry in Ceylon.

The Lady Norah Hodgson, in a short speech, declared the hospital open, and wished it success. The key with which it had been opened, she said, was an ordinary one, but it must not be forgotten that a golden key also was necessary if it was to be maintained with efficiency in the future.

Sir Henry Burdett said it was a great pleasure to him to be present at the opening of an institution which emphasised a departure in the treatment of disease sufficient to make it memorable and of public importance. The treatment of chronic cases like hip disease and spinal complaint was very costly; it involved an enormous amount of time to get any result, and the consequence was that an institution of this kind, rightly conducted, was very expensive. By strictly limiting the admissions to children of parents whose circumstances were such that they could not at home provide the necessary treatment (without which the child must perish from disease preventible by removal to a building like this), they brought home to parents the fact that every person was given the opportunity of testifying whether they were really living a thankful life when they were provided with more than they needed of God's good gifts. They could avoid settling down to the ease and rest of riches which were enervating to body and soul. Here in a rich district like this it must be a blessing to many people to be given the opportunity of living quietly and taking an interest in perhaps only one little sufferer, and so learning how to suffer and how to live. He spoke as one who had resided in hospitals for more years probably than almost any other man. He was familiar with the various forms of suffering entailed on humanity, and he knew that after being in the general wards it was always a rest to go into the children's ward. By taking an interest in them, and by observing how a little child could suffer, we may learn to be thankful for the blessings of health; such an opportunity of helping ourselves and others was afforded by an institution like this.

### Some Paying Institutions.

Now it was with sorrow that he said it, but it was a fact, that work—successful, growing, and developing work—on a basis of payment by patients such as they had before them to-day, and at which they had met to rejoice, had an evil counterpart elsewhere at the present day. It sometimes happened in so-called "Homes" that the medical service and arrangements were not what they should be in cases where the owners of the homes were aided by the callousness of parents who in his judgment unnecessarily added to the sufferings of their children. That was a very sad statement, but one that was borne into his mind from visits of inspection which he had paid to some so-called institutions which received payment, and he was convinced that it was not right to allow it to be possible for anyone to open an institution of the kind and to exact payment unless it was open to be inspected. There were institutions by which much injury might be wrought. He would give one or two instances which had come under his immediate notice in private-venture homes and orphanages. In one some children had not for years been visited by their parents, and some had been tied down in bed in the same position for two years. No visits had been paid by surgeons of advanced science, and the only visitor was often a local doctor, and then only when sent for by the authorities. That was the case in one institution. It should be looked into and altered. Next take the case of an orphanage; he had one in his eye where there were 250 children, and the boast was that the inclusive annual cost per child was less than £20 per annum. At what price? The unnecessary suffering and injury of defenceless children! A large proportion of the children were girls between five and 15 years of age. They did the work of the whole institution, including house-work, scrubbing the stone staircases, making all the socks, stockings, and underclothing, and this was done to make a show, at the children's expense, of the economical results. It was under the management of a London and a local committee, and they were constantly in and out, but they seemed oblivious to a child's limited capacity. So these committees were conducting an establishment of which, if they realised its cruelties, there was not a man or woman on the committee who would not be ashamed. Another case. For 18 years it had gone on, and it was very difficult to get an inside inspection of it. It was in the heart of the most fashionable part of London. There were upwards of 20 girls, daughters of officers or professional men, and not only was there a lack of comfort and an absence of proper means of obtaining books and education, but the sanitary and washing arrangements were bad, and there was a lack of warmth and means of exercise. As a result of his visit that establishment had ceased to exist as an institution, but those who conducted it had again started in the country, and it would hardly be believed, but it is the fact that the majority of the parents had sent the children back to the same injurious, dirty conditions which the children had suffered from in the first instance. What a contrast the Sevenoaks Hospital presented with its ever-loving care and treatment of the child inmates!

### The Inspection and Registration of Hospitals.

"We want," the speaker went on, "inspection in these private adventure homes, or so-called institutions conducted for profit by a nominal committee or by private proprietors. The following are the heads of inquiry which I would suggest to ensure that any inspections made are effective for good. The inspector should ascertain: (1) The number of years they have been at work; (2) (*a*) The extent to which paying patients are received from whom a profit is made, and (*b*) other patients paying a smaller rate not remunerative; (3) The extent to which restraint is practised at these institutions; (4) Whether any accounts are published of the profits made by the reception of paying patients, or whether all profits are taken by the lady superintendent or proprietor, who pays the expenses and takes all they can make out of this system; (5) the size of the rooms in which the children are kept, the amount of cubic space in each, the number of years they are kept in the institution, and a statement of the results of all the cases which have been under treatment from the outset; (6) whether the homes are in connection with existing hospitals under the charge of (*a*) a responsible surgeon attached to some great general hospital, or (*b*) in the care of general practitioners in the neighbourhood; (7) whether any payment is made for the medical services; (8) whether the sisters or nurses are paid in whole or in part, their rates of remuneration, and the sources from which they are derived; (9) whether the institutions are ever inspected by anybody, and to what extent the children are visited, if at all, by their friends, and the regulations for such visits. We want expert inspectors for all institutions where children are treated or relieved. These inspectors should be trained for the work, and care should be taken that they pursue their inquiries not in an inquisitorial way, but with the view to show the proprietors and committees how and where things can be improved and put right. The fact is, in my judgment, that there is need for fewer generalities in the reports, and the substitution of definite information, conveying an accurate knowledge of the state of health and discipline maintained. As matters are at present, sometimes the founder or founders of the home are sole managers, sometimes there is the nominal assistance of a committee, and sometimes in the larger institutions there is more than one committee; but they have never changed the system because the individual members lack the initiative to look at matters as they in reality are, and to bring about great changes in the methods pursued with a view to secure the better health and protection of the weaker children especially, and to provide that nothing in the system tends to injure but only to build up the moral and physical life of each child. At the present time the public has practically no choice but to place unbounded confidence in the character of those in charge. No one in reality can tell whether the facts justify that confidence or not, and it is in the interests of the managers, the inmates, and the subscribers alike, that we should have a regular Governmental inspection of all institutions which take public money or are conducted for profit made out of patients' payments. There can be no question of the value of the help which such skilled supervision would afford to all who are conscientiously striving to render their homes as efficient as possible. I, further, have advocated for years, and shall look to the present Home Secretary, Mr. Akers-Douglas, to provide for the compulsory registration of homes. It should be made illegal for anyone to set up such a home without first securing a certificate of registration, for the latter would not be granted without satisfactory proofs of the *bona fides* of the undertaking. It is a fact that unless inspection and registration of these homes—and I believe of all institutions—is brought about, we may have, as one result of the war (owing to the number of buildings erected for convalescent homes, and not needed), a great epidemic of the private-venture institutions for the adventurer's benefit. I have spoken about the necessity of having responsible surgeons attached to these institutions, and I rejoice that I am to be followed by an eminent surgeon, to whom you are indebted for his work here. I hope he will follow up what I have said by giving his views with reference to the restraint of children in one constrained position for years, and say whether he considers that, having regard to the results which are possible, the chronic sufferers from hip disease should be treated in an institution of this kind, instead of being dealt with by the surgeon's knife, the results of which are often more satisfactory and more rapid.

£700 as a Thankoffering.

"You have heard from the chairman that you need £700. That is a sum so small in wealthy Sevenoaks that I am perfectly certain that it has only to be known, from a visit to this institution, what are the benefits gained here by children, for the money to come flowing in during the next week. It should be impressed upon the people of Sevenoaks, not only as a matter of education, but also in their own interest, the advantages of studying the history of this institution and that of the Alexandra Hospital in London, which are models of efficiency in their management and arrangements. There is an opportunity to give, if you come quickly with your cheques, at the beginning of this new week in its history. There is a child who has been here four years—you, who are older, and have rheumatism, and other ills, think of this. The surgeons hope that child will recover, and, like the young soldier in Ceylon, live to be a healthy wage-earner. Is there anyone with good means residing in Sevenoaks who can, from the point of view of the plains of Heaven, refuse to help? I hope this institution may extend and endure, and that Miss Jackson, and all those who have helped her, may realise something of the glorious results they have been able to bring about, by their own belief in the possibilities of this hospital, on behalf of the children who would otherwise still be in suffering and misery, if not in their graves. I am one of those who believe that there is a great and extending work in this country for women; I always rejoice to see women taking part in charitable work, and in an institution of this kind a woman is better than a man. I conclude by proposing a very hearty vote of thanks to the Lady Norah Hodgson for her kindness in opening this hospital."

No Room in the General Hospitals.

Mr. W. J. Walsham, F.R.C S., said he had been asked to say a few words, from his own point of view as surgeon to a large London hospital and consulting surgeon to the Sevenoaks one, as to the benefits to the poor of having an institution like this. There were a few points which he would like to mention. In the London hospitals, he was sorry to say, these cases had often to be refused, because they were very chronic, and there were always hundreds of acute cases seeking admission. There might be a poor child in the early stage of hip disease, but there was also the bread-winner, perhaps suffering from malignant cancer, and the child had to wait. Thus the time was lost. And then these children occupied a bed so long. He held that it was better to give them rest, and to do without the surgeon's knife, and this was possible if they had sufficient time.

"I can only say," Mr. Walsham continued, and the statement was received with enthusiastic applause, "that if I had a child with hip disease, and Miss Jackson would take charge of it, I would send it here." It was impossible, he went on, to keep these cases in a general hospital, where a hundred and one other cases were calling upon the doctors for room, and they had to be sent home, apparently cured, but with the seeds of disease in them, and often an operation was called for later on. They knew at Sevenoaks what it was to have children sent away after an operation which had appeared to be successful. What happened? Sooner or later they returned, practically incurable. Then, a London hospital was not the best place for poor children suffering from tuberculous disease. He felt there was a great need for a few free beds. As they all knew, owing to the financial conditions, a small amount had to be charged, but he was sure it was more than many poor people could afford. He could speak, after the time he had been attached to the hospital, of the benefits received there. The children could not have better attention or skill from the local doctors, who were always ready to meet the consulting surgeons, and as for those dear women, Miss Jackson and Miss Rose, he could not praise them too much. It only required £1,000 to endow a bed. What was £100,000 in a place like Sevenoaks? Surely someone would come forward to endow some beds.

A vote of thanks to the architect, Mr. Graham Jackson, R.A., and a song which was beautifully rendered by Miss Florence Monk moved the audience greatly and concluded this part of the ceremony. A short service of dedication was held in the other large ward, and during the evening the building was open to the public, and a series of concerts was given. The amount collected at the opening was about £63.

The New Building.

The new building, of which the foundation stone was laid in June, 1901, is on a magnificent site on Tub's Hill, a few minutes' walk from Sevenoaks (Tub's Hill) Station, in a very airy position, and so placed that all the benefits of sunshine may be felt. It faces south-west and north-east, and at either end, opening from the two large wards, is a balcony. The administrative block is in the middle, and is flanked by the two large and two small wards. On the top floor is a small ward, which, with bath-room and nurses' room, can be isolated by means of a carbolic sheet hung across the passage, in case of infection. The servants' bedrooms are also on this floor, commanding a splendid view of the country round. On the first floor are the matron's room and the bedrooms of the staff nurse and the four lady probationers, who at present, with Miss Jackson and Miss Rose, comprise the nursing staff; and the linen-room. The committee-room, dining-room, surgery (with X-rays), dispensary, etc., are on the entrance floor; while on the basement are the servants' hall, kitchen, pantry, meat larder, milk larder, and splint-room. The building is heated by a central hot-air apparatus situated in the basement, the air being diffused in wards and passages by central radiators. There is a lift for dinners, etc. There is no colouring on the walls, and time is to be allowed for drying. The wards and passage on the entrance floor are of Asbolith flooring. The building is designed for 34 beds, and can be added to in future if desired. The builders are Messrs. Wise & Sons.

It was intended to move the patients from the old hospital on "The Vine" in the course of a few days.

